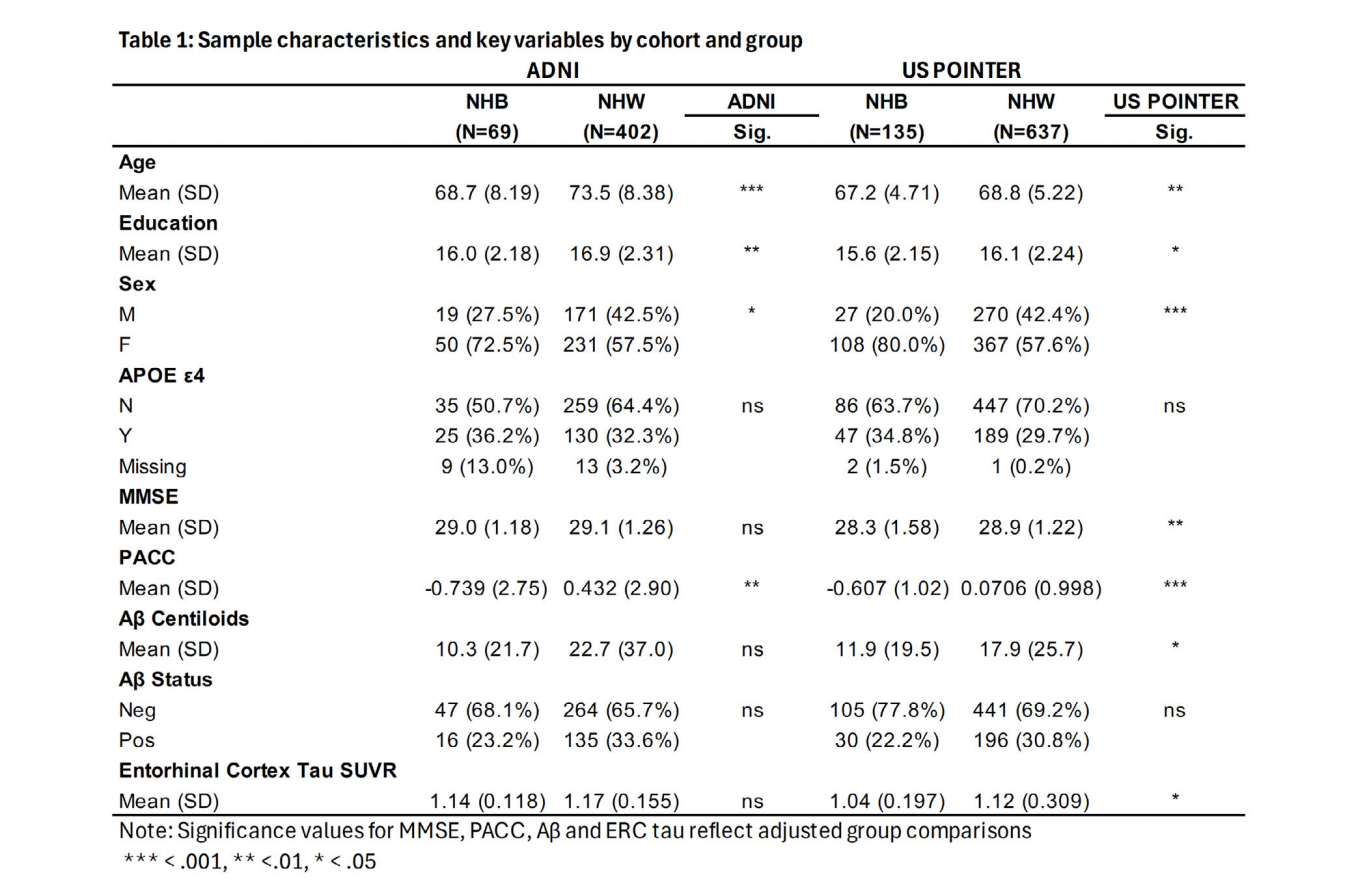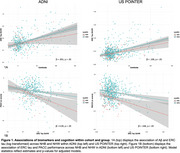# Evaluation of Alzheimer's disease biomarkers and cognition in non‐Hispanic Black and non‐Hispanic White participants in ADNI and US POINTER Imaging

**DOI:** 10.1002/alz70856_100136

**Published:** 2025-12-25

**Authors:** Talia L. Robinson, Theresa M. Harrison, Kathryn V Papp, Laura D Baker, Heather M Snyder, Prashanthi Vemuri, Charles Decarli, Rebecca E. Amariglio, Susan M. Landau, US POINTER Study Group, Alzheimer's Disease Neuroimaging Initiative

**Affiliations:** ^1^ Massachusetts General Hospital, Boston, MA, USA; ^2^ Neuroscience Department, University of California, Berkeley, Berkeley, CA, USA; ^3^ Brigham and Women's Hospital, Boston, MA, USA; ^4^ Wake Forest University School of Medicine, Winston‐Salem, NC, USA; ^5^ Alzheimer's Association, Chicago, IL, USA; ^6^ Department of Radiology, Mayo Clinic, Rochester, MN, USA; ^7^ Department of Neurology & Imaging of Dementia and Aging Laboratory, University of California Davis, Sacramento, CA, USA

## Abstract

**Background:**

Given low representation of diverse populations in Alzheimer's disease (AD) research, it remains unclear if AD biomarker relationships observed primarily in non‐Hispanic White (NHW) cohorts generalize to other groups. Here we evaluate associations between key AD biomarkers and cognitive performance among cognitively unimpaired non‐Hispanic Blck (NHB) and NHW participants across 2 cohorts with unique sample characteristics.

**Method:**

Participants included cognitively unimpaired NHB and NHW participants with Aβ and tau PET in ADNI (69 NHB, 402 NHW) and POINTER Imaging (135 NHB, 637 NHW). We examined harmonized measures of global Aβ (in centiloids), entorhinal cortex (EC) tau (quantified with MK6240 in POINTER and FTP in ADNI) and Preclinical Alzheimer's Cognitive Composite (PACC) scores. Regression models within each ethnoracial group evaluated relationships between 1) Aβ and EC tau, and 2) EC tau and PACC performance. Interaction models within each cohort were also conducted to assess differences in these associations by ethnoracial group. All models were adjusted for age and gender. Models with PACC additionally adjusted for education.

**Result:**

In US POINTER, NHBs had lower Aβ and EC tau burden compared to NHWs (Table 1). In ADNI, there was no difference in EC tau between groups, and no differenence in Aβ after adjusting for age and gender. In US POINTER, the positive Aβ‐EC tau relationship was observed in both ethnoracial groups whereas in ADNI, Aβ was associated with higher EC tau in the NHW but not the NHB group. In both cohorts, EC tau was associated with worse PACC performance in both NHB and NHW and there were no differences in this association by ethnoracial group in interaction models (Figure 1).

**Conclusion:**

NHBs had lower Aβ and EC tau than NHWs in POINTER but not ADNI, and there were inconsistent associations between Aβ and EC tau across cohorts. Despite these differences, AD biomarkers appear similarly related to cognitive outcomes across ethnoracial groups in both cohorts. Future analyses will attempt to further determine the generalizability of AD biomarker relationships in diverse samples by considering matching on relevant health and demographic characteristics, and inclusion of additional cohorts with harmonized biomarker and cognitive measurements.